# Intracellular Ca^2+^ Signalling in the Pathogenesis of Acute Pancreatitis: Recent Advances and Translational Perspectives

**DOI:** 10.3390/ijms21114005

**Published:** 2020-06-03

**Authors:** Petra Pallagi, Tamara Madácsy, Árpád Varga, József Maléth

**Affiliations:** 1 First Department of Medicine, University of Szeged, H6720 Szeged, Hungary; pallagi.petra@med.u-szeged.hu (P.P.); tamaramadacsy@gmail.com (T.M.); varga.arpad9105@gmail.com (Á.V.); 2 HAS-USZ Momentum Epithelial Cell Signaling and Secretion Research Group, University of Szeged, H6720 Szeged, Hungary; 3 HCEMM-SZTE Molecular Gastroenterology Research Group, University of Szeged, H6720 Szeged, Hungary

**Keywords:** acute pancreatitis, Ca^2+^ signalling, bile acid, acinar cell necrosis, epithelial ion transport

## Abstract

Intracellular Ca^2+^ signalling is a major signal transductional pathway in non-excitable cells, responsible for the regulation of a variety of physiological functions. In the secretory epithelial cells of the exocrine pancreas, such as acinar and ductal cells, intracellular Ca^2+^ elevation regulates digestive enzyme secretion in acini or fluid and ion secretion in ductal cells. Although Ca^2+^ is a uniquely versatile orchestrator of epithelial physiology, unregulated global elevation of the intracellular Ca^2+^ concentration is an early trigger for the development of acute pancreatitis (AP). Regardless of the aetiology, different forms of AP all exhibit sustained intracellular Ca^2+^ elevation as a common hallmark. The release of endoplasmic reticulum (ER) Ca^2+^ stores by toxins (such as bile acids or fatty acid ethyl esters (FAEEs)) or increased intrapancreatic pressure activates the influx of extracellular Ca^2+^ via the Orai1 Ca^2+^ channel, a process known as store-operated Ca^2+^ entry (SOCE). Intracellular Ca^2+^ overload can lead to premature activation of trypsinogen in pancreatic acinar cells and impaired fluid and HCO_3_^-^ secretion in ductal cells. Increased and unbalanced reactive oxygen species (ROS) production caused by sustained Ca^2+^ elevation further contributes to cell dysfunction, leading to mitochondrial damage and cell death. Translational studies of AP identified several potential target molecules that can be modified to prevent intracellular Ca^2+^ overload. One of the most promising drugs, a selective inhibitor of the Orai1 channel that has been shown to inhibit extracellular Ca^2+^ influx and protect cells from injury, is currently being tested in clinical trials. In this review, we will summarise the recent advances in the field, with a special focus on the translational aspects of the basic findings.

## 1. Ca^2+^ Signalling and Acute Pancreatitis

Acute pancreatitis (AP) is an inflammatory disease of the pancreas associated with significant morbidity and mortality. AP is one of the most frequent causes of hospitalisation among non-malignant gastrointestinal disorders, whereas the global disease incidence has increased in the past several decades [1,2,3]. Although overall disease mortality has decreased in recent years [4], the mortality from severe forms of AP (comprising about 10% of all cases) remains remarkably high (~28%) [5]. AP is primarily caused by impacted gallstones or heavy alcohol consumption; however, the incidence of iatrogenic AP caused by endoscopic retrograde cholangiopancreatography (ERCP) or drug administration (such as L-asparaginase) has also increased [5]. Regardless of the different pathogenic factors that may lead to acute inflammation of the pancreas, all cases are associated with sustained elevated intracellular Ca^2+^ levels, which are a hallmark of AP pathogenesis [6]. Intracellular Ca^2+^ overload can lead to premature trypsinogen activation [7,8], mitochondrial damage and cell necrosis in pancreatic acinar cells [9]. Our group has reported that sustained elevation of intracellular Ca^2+^ in ductal cells impairs fluid and HCO_3_^-^ secretion [10,11], which are key functions of ductal cells, and also triggers mitochondrial damage with consequent ATP depletion and cell damage [12]. As detailed below, toxins induce Ca^2+^ release from the endoplasmic reticulum (ER) intracellular Ca^2+^ stores, which is considered as the initial step in the development of sustained Ca^2+^ elevation. Inhibition of the uncaged IP_3_-induced Ca^2+^ release by caffeine and other non-xanthine phosphodiesterase inhibitors leads to the impairment of toxin-induced Ca^2+^ release and prevents mitochondrial depolarisation and acinar cell necrosis. It also improves the severity of cerulein, bile acid or fatty acid ethyl ester (FAEE)-induced experimental AP in mice [13]. Sustained Ca^2+^ signals can lead to mitochondrial injury, which can activate both apoptosis and necrosis. In the pathogenesis of AP opening of the mitochondrial permeability transition pore (MPTP) triggered by sustained elevated Ca^2+^ levels is an initiating step in mitochondrial membrane potential (ΔΨ_m_) loss, impaired mitochondrial ATP synthesis and increased permeability of the inner mitochondrial membrane, resulting in mitochondrial swelling and necrosis [14,15]. Genetic or pharmacologic inhibition of the MPTP resulted in the improvement of the AP phenotype and acinar [16] and ductal [17] cell injury both in vitro and in vivo.

### 1.1. Intracellular Ca^2+^ in Biliary AP

**Figure 1:**
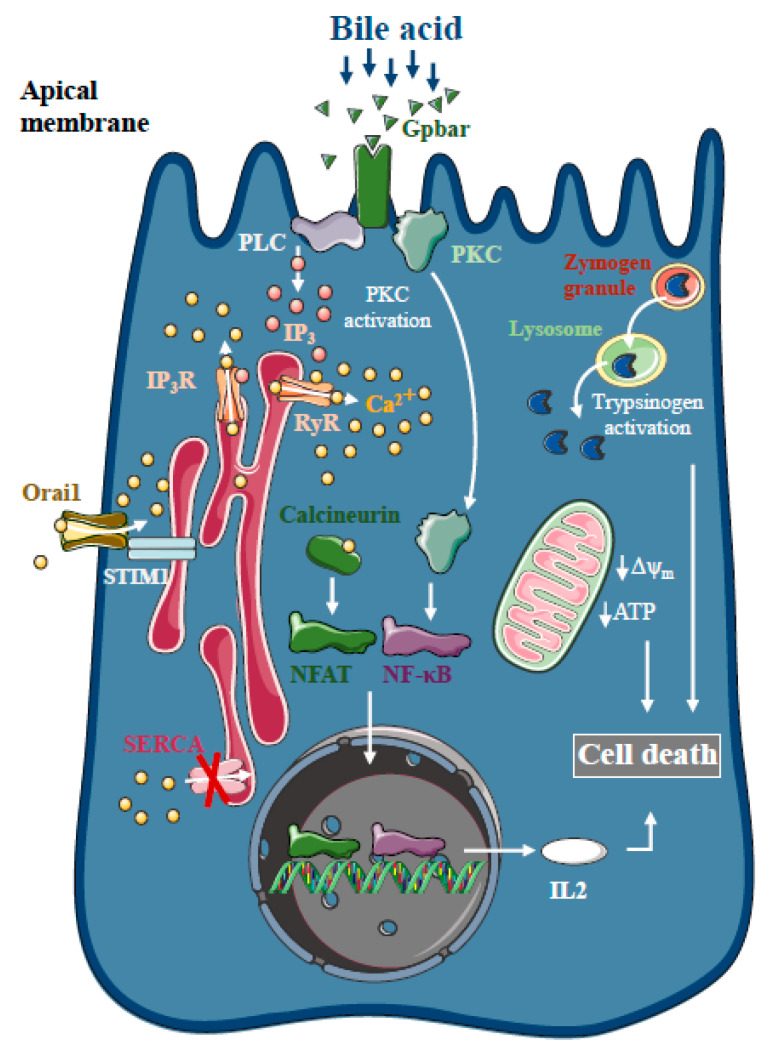
Intracellular Ca^2+^ signalling in biliary acute pancreatitis. Bile acids dose-dependently release Ca^2+^ from intracellular stores via activation of IP_3_ and ryanodine receptors (RyR). The inhibition of the sarco-endoplasmic reticulum Ca^2+^ pump (SERCA) and activation of Orai1-mediated extracellular Ca^2+^ influx contributes to the sustained global Ca^2+^ signals. Bile acids can activate the G-protein-coupled cell surface bile acid receptor (Gpbar) at the apical membrane of pancreatic acinar cells that also release Ca^2+^ from the endoplasmic reticulum. Mitochondrial Ca^2+^ overload can lead to mitochondrial damage by opening the mitochondrial permeability transition pore and dissipating the mitochondrial membrane potential. In addition, bile acids have been demonstrated to activate calcineurin in a Ca^2+^-dependent manner in pancreatic cells, leading to premature digestive enzyme and NF-κB activation.

Bile acids are known to trigger dose-dependent elevations in intracellular Ca^2+^ concentrations in isolated pancreatic acinar [18] and ductal cells [19] in vitro, which is due to Ca^2+^ release from intracellular stores via the activation of IP_3_ and ryanodine receptors, sarco-endoplasmic reticulum Ca^2+^ pump (SERCA) inhibition [20] and extracellular Ca^2+^ influx activation [21] (Figure 1). The toxic effects of bile acids on pancreatic acinar cells also involve the activation of the G-protein-coupled cell surface bile acid receptor (Gpbar1) at the apical membrane, which also contributes to the development of sustained Ca^2+^ elevation and its downstream effects [22]. On the other hand, Gpbar1 knockout mice were protected against taurolithocholic acid 3-sulfate-induced AP, an experimental model of biliary AP.

The pancreatic ductal epithelia can be another possible target of bile acids in the exocrine pancreas. Previous reports have suggested that increases in intracellular Ca^2+^ in pancreatic ductal epithelial cells leads to a marked dose-dependent decrease in HCO_3_^-^ secretion upon bile acid exposure of isolated pancreatic ductal fragments [19]. In addition, bile acids inhibit intracellular ATP production and decreased ΔΨ_m_ in acinar [23,24] and ductal cells [12]. Interestingly, bile acid toxicity was not abolished by the removal of intracellular Ca^2+^ elevation using a Ca^2+^ chelator, BAPTA-AM, neither in acinar [23] nor in ductal cells [19], suggesting the existence of other parallel Ca^2+^-independent effects of bile acids on the mitochondria. Besides inducing mitochondrial toxicity, bile acids have also been shown to activate calcineurin in a Ca^2+^-dependent manner in pancreatic cells, leading to premature digestive enzyme and nuclear factor kappa-light-chain-enhancer of activated B cells (NF-κB) activation [25]. The inhibition of calcineurin pharmacologically or by genetic knockout, reduced the severity of taurolithocholate 3-sulfate (TLCS)-induced AP and impaired protein kinase C, an upstream regulator of NF-κB activation and translocation [26].

### 1.2. Intracellular Ca^2+^ in Alcoholic AP

**Figure 2:**
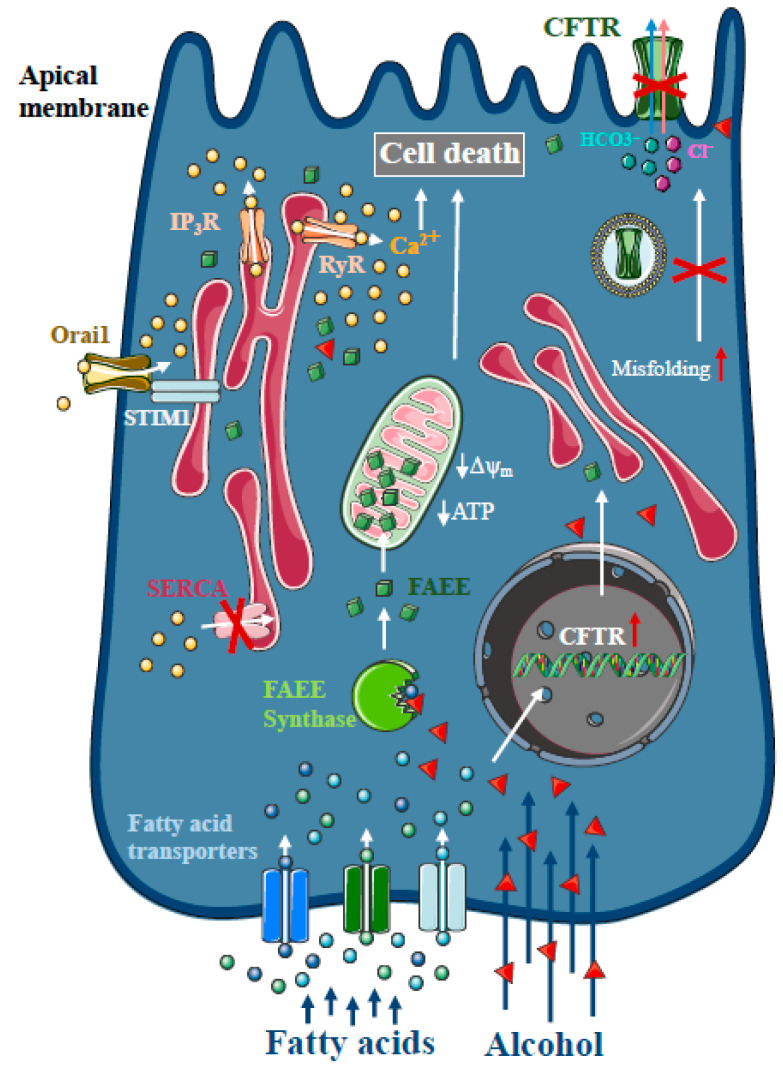
Intracellular Ca^2+^ signalling in alcoholic acute pancreatitis. In the pancreas, non-oxidative ethanol metabolism is the dominant metabolic pathway mediated by enzymes with fatty acid ethyl ester (FAEE) synthase activity, which combine ethanol and fatty acids to generate FAEE. In pancreatic acinar cells, FAEEs are accumulated in the mitochondria and their local breakdown leads to localised high concentrations of fatty acids. Similar to bile acids, FAEEs induce sustained [Ca^2+^]_i_ elevation and drop of cellular ATP leading to necrosis. In addition, alcohol and fatty acids inhibit fluid and HCO_3_^-^ secretion in the pancreatic ductal epithelia, mainly due to the impaired expression and function of the cystic fibrosis transmembrane conductance regulator (CFTR).

Heavy alcohol consumption is the second most frequent cause of AP [1]. As only a minority of alcoholics develop AP, genetic factors seem to play a major role in disease pathogenesis [27]. However, direct treatment with ethanol and different non-oxidative ethanol metabolites have a damaging effect on acinar and ductal cells. In contrast to other organs, such as the liver, non-oxidative ethanol metabolism is the dominant metabolic pathway in the pancreas [28], which is mediated by enzymes with FAEE synthase activity [29], which combine ethanol and fatty acids to generate FAEE [30]. Early studies suggested that more than 70% of FAEEs are preferentially accumulated in the mitochondria in cardiomyocytes, which is the site of fatty acid hydrolysis [31]. Huang et al. investigated FAEE hydrolysis in pancreatic acinar cells using a fluorescently tagged palmitoleic acid probe that releases fluorescein upon hydrolysis [32]. Their results suggest that FAEEs accumulate in mitochondria and that their local breakdown leads to high concentrations of fatty acids. Like bile acids, FAEEs have been shown to induce sustained [Ca^2+^]_i_ elevation and reduced levels of cellular ATP, leading to necrosis [9,33,34] (Figure 2). Our group has demonstrated previously that alcohol and fatty acids inhibit fluid and HCO_3_^-^ secretion in the pancreatic ductal epithelia, mainly due to impaired expression and function of the cystic fibrosis transmembrane conductance regulator (CFTR) [35], which was restored by ATP supplementation [36]. Inhibition of CFTR activity was mediated by sustained intracellular Ca^2+^ elevation, decreased adenosine 3′,5′-cyclic monophosphate (AMP) levels and impaired ATP production accompanied by ΔΨ_m_ depolarisation. Ethanol has reduced CFTR expression via accelerated plasma membrane turnover and impaired CFTR membrane stability. These alterations in the ductal cells increased the severity of alcohol-induced AP in mice.

### 1.3. Intracellular Ca^2+^ in Drug-Induced AP

AP is a relatively frequent complication of medical treatments, leading to painful inflammation and hospitalisation, and may serve as an indication to alter or cease otherwise effective therapy. Asparaginase, a long-term medication used to treat acute lymphoblastic leukaemia (ALL) in children, is one of the most likely drugs to induce AP. The incidence of asparaginase-induced AP is above 10% [37], making it one of the most common causes for halting therapy for ALL [38]. Recently, Peng et al. demonstrated that the treatment of isolated pancreatic acinar cell clusters with asparaginase triggers similar changes in intracellular Ca^2+^ signalling as bile acids and ethanol metabolites [39]. They showed that asparaginase treatment leads to intracellular Ca^2+^ release, followed by extracellular Ca^2+^ entry activation. Ca^2+^ extrusion was also severely impaired, due to decreased intracellular ATP production, leading to cell necrosis. The inhibition of protease-activated receptor 2 (PAR2) abolished the toxic intracellular Ca^2+^ signals, suggesting that the toxic effect of asparaginase is mediated by PAR2 activation. In addition, inhibition of the Ca^2+^ entry by the selective Orai1 inhibitor, GSK-7975A, protected acinar cells from Ca^2+^-induced damage. Similar effects were observed in vitro and in vivo by replacing glucose with galactose, which prevented the loss of ATP and protected acinar cells from necrosis [40].

## 2. Interplay between Ca^2+^ and Redox Signalling in Acute Pancreatitis

Reactive oxygen species (ROS) are normal components of physiological signalling processes in cells. The intracellular level of ROS is balanced by oxidant production and antioxidant processes. ROS are generated during mitochondrial respiration and are mainly derived from complex I and III of the mitochondrial electron transport chain [41]. In recent decades, numerous roles of ROS have been identified to play a variety of roles in intracellular signalling [42]. Booth et al. demonstrated that the ER–mitochondrial interface hosts a H_2_O_2_ nanodomain, which is triggered by increased cytoplasmic Ca^2+^ levels and is a positive regulator of Ca^2+^ oscillations. Such nanodomains can be considered as important elements in inter-organelle communication [43]. ROS production also exerts various effects on the ion channels and pumps that are involved in intracellular Ca^2+^ signalling, which was reviewed in detail previously [44]. Although ROS play an important role in cellular signalling, unbalanced ROS production contributes to the pathogenesis of a variety of diseases by disrupting the lipid membranes, proteins and nucleic acids [45]. In relation to AP, previous studies have shown that ROS generation determines pancreatic acinar cell fate, as elevated levels of ROS induced by the oxidant menadione led to increased apoptotic cell death [46]. In addition, bile acids have been shown to induce ROS generation within the mitochondria in pancreatic acinar cells, leading to mitochondrial Ca^2+^ increases in both mice and humans [47], which was inhibited by *N*-acetylcysteine. Therefore, mitochondrial targeting of antioxidants may be a potential therapeutic strategy for treating AP. However, mitochondrial targeting of antioxidants using positively charged molecules that insert into the inner mitochondrial membrane was harmful to pancreatic acinar cells, most likely due to the wide involvement of ROS in the regulation of intracellular signalling and bioenergetics [48]. Moreover, during AP pathogenesis, ROS, released by circulating neutrophils during the inflammatory response, might also contribute to the development of cell damage and local and systemic complications of AP [49].

Transient Receptor Potential Melastatin-like 2 (TRPM2) is a Ca^2+^-permeable non-selective cation channel, which has been identified to act as a cellular redox sensor [50,51], and plays an important role in physiological processes such as insulin secretion [52] and the central regulation of body temperature [53]. Perraud et al. demonstrated that TRPM2 is activated by H_2_O_2_ via an indirect mechanism mediated by the intracellular production of adenosine diphosphate ribose (ADPR) which then binds to and stimulates the C-terminal ADPR pyrophosphatase Nudix-like domain (NUDT9-H motif) of TRPM2 [54]. This channel not only has prominent roles in physiology, but its contribution to pathologic conditions was also emphasised [55]. Ca^2+^ influx mediated by TRPM2 elevates chemokine production in monocytes, resulting in increased neutrophil infiltration in inflammatory bowel diseases [56]. In addition, TRPM2 contributes to the pathogenesis of xerostomia induced by irradiation [57]. Liu et al. demonstrated that ROS production increases as a side effect of irradiation during radiotherapy of head and neck cancers, leading to TRPM2 activation, resulting in extracellular Ca^2+^ influx in salivary glands. Sustained intracellular Ca^2+^ elevation reduced the secretory function of acinar cells, leading to the development of xerostomia, which is a common side effect of radiotherapy in these patients. In addition, after irradiation, mitochondrial Ca^2+^ concentration and ROS production are elevated in a TRPM2-dependent manner, leading to impaired Δψ_m_ and activated caspase-3 [58]. These changes result in a sustained decrease in stromal interaction molecule 1 (Stim1) expression and decreased store-operated Ca^2+^ entry (SOCE). In the endocrine pancreas, TRPM2 plays a role in diabetic stress-induced mitochondrial fragmentation. Abuarab et al. demonstrated that high glucose concentrations stimulate ROS production, which activates TRPM2, leading to lysosomal membrane permeabilisation and Zn^2+^-mediated mitochondrial fission [59]. These studies highlight that TRPM2 is expressed in various epithelial cells and they demonstrate the fundamental role of this protein in the pathogenesis of oxidative-stress-related disease pathogenesis. However, the expression or function of TRPM2 in exocrine pancreatic cells has not been investigated.

**Figure 3:**
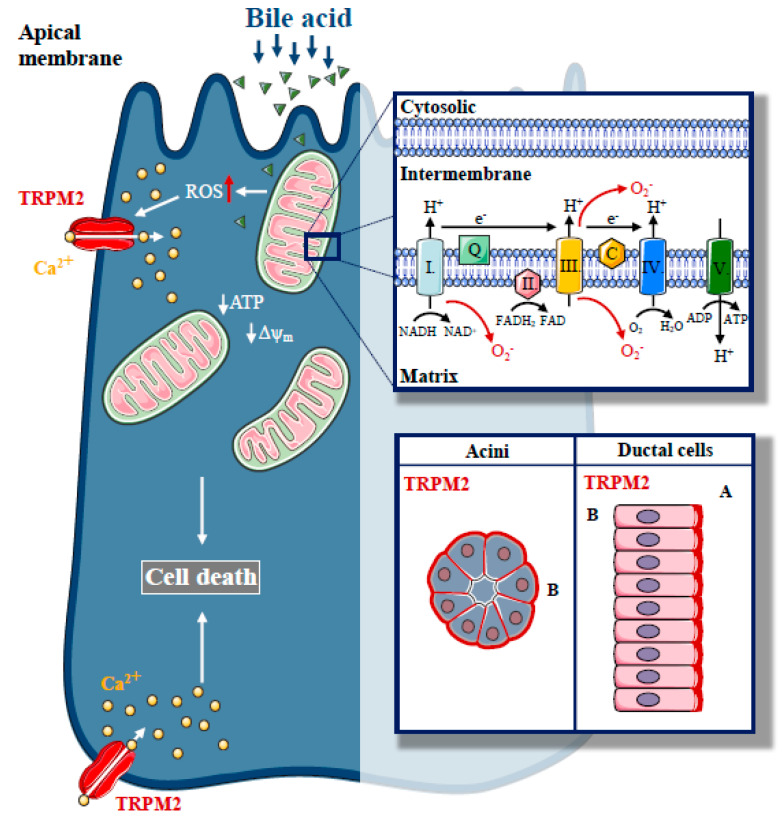
The role of Transient Potential Melastatin-like 2 (TRPM2) in biliary pancreatitis. Increased, unbalanced production of ROS, which are generated during physiological mitochondrial respiration, are mainly derived from complexes I and III of the mitochondrial electron transport chain, a crucial event in the pathogenesis of biliary acute pancreatitis. In our recent study, we described the expression of a redox sensitive cation channel, TRPM2, in the exocrine pancreas with basolateral localisation in acinar cells and apical localisation in ductal cells. H_2_O_2_-induced oxidative stress activated TRPM2, whereas TRPM2 knockout decreased the bile acid-induced Ca^2+^ elevation in acinar cells and prevented acinar cells from bile-acid-induced necrosis. Genetic deletion of TRPM2 reduced the severity of bile-acid-induced experimental pancreatitis.

In a recent study, we described the expression of TRPM2 in the exocrine pancreas and established the role of TRPM2 in biliary AP [60] (Figure 3). TRPM2 was localised to the basolateral membrane in mouse pancreatic acinar cells, but apically in mouse pancreatic ductal cells. We have found that H_2_O_2_-induced oxidative stress activated TRPM2. In our experiments, CDC resulted in [Ca^2+^]_i_ elevation, both in acinar and ductal cells, but the knockout of TRPM2 decreased the CDC-induced Ca^2+^ elevation only in acinar cells, suggesting that the TRPM2 channel contributes to approximately 22% of the bile-acid-generated Ca^2+^ signal in acinar cells. Interestingly, our results demonstrated that bile-acid-induced intracellular ROS production is remarkably different in pancreatic acinar and ductal cells. These findings provide a mechanistic explanation for the differing involvement of TRPM2 in the Ca^2+^ response generated by bile acid in these different cell types. The difference in ROS production may be explained by the difference in the mitochondrial mass in acinar versus ductal cells [12,61]. As expected, we were not able to detect any protective effects due to genetic deletion of TRPM2 in the inhibition of pancreatic ductal secretion induced by bile acids. In this study, the lack of TRPM2 significantly reduced the H_2_O_2_ and bile-acid-induced necrosis in pancreatic acinar cells. We also demonstrated that the rate of necrosis was significantly reduced in an experimental model of biliary pancreatitis in TRPM2 knockout mice as compared to wild type (WT) animals, consistent with in vitro observations. In agreement with our findings, Booth et al. documented that incubation of pancreatic acinar cells with TLCS in vitro leads to Ca^2+^-dependent necrosis, which is abolished by pre-treatment with BAPTA-AM [47]. Using different inhibitors to prevent apoptosis and necrosis, the authors suggested that elevated intracellular and intramitochondrial ROS are the major triggers of apoptosis, while increases in intracellular and intramitochondrial Ca^2+^ are the major triggers of necrosis. Bile acids are known to inhibit cellular ATP production [24] and decrease ΔΨ_m_ [23]. Thus, we also measured the effect of bile acid treatment on ΔΨ_m_ in TRPM2 KO and WT acinar cells. Genetic deletion of TRPM2 or removal of extracellular Ca^2+^ markedly reduced the decrease in Δψ_m_, suggesting that TRPM2-mediated extracellular Ca^2+^ influx plays a crucial role in oxidative-stress-induced mitochondrial damage. In contrast, this protective effect was not detected in bile-acid-treated cells. This may be explained by the direct Ca^2+^-independent mitochondrial toxicity of bile acids [62]. Based on the previous findings of our group [19] and others, [23] we hypothesise that prevention of intracellular Ca^2+^ elevation cannot completely abolish the toxic effects of bile acids. We did not observe mitochondrial fragmentation in our experiments, which has been previously linked to TRPM2 activation [59]. In addition, genetic deletion of TRPM2 reduced the severity of bile-acid-induced experimental pancreatitis; however, we did not observe this protective effect in cerulein-induced AP in TRPM2 knockout mice.

## 3. Store-Operated Ca^2+^ Entry and Acute Pancreatitis

**Figure 4:**
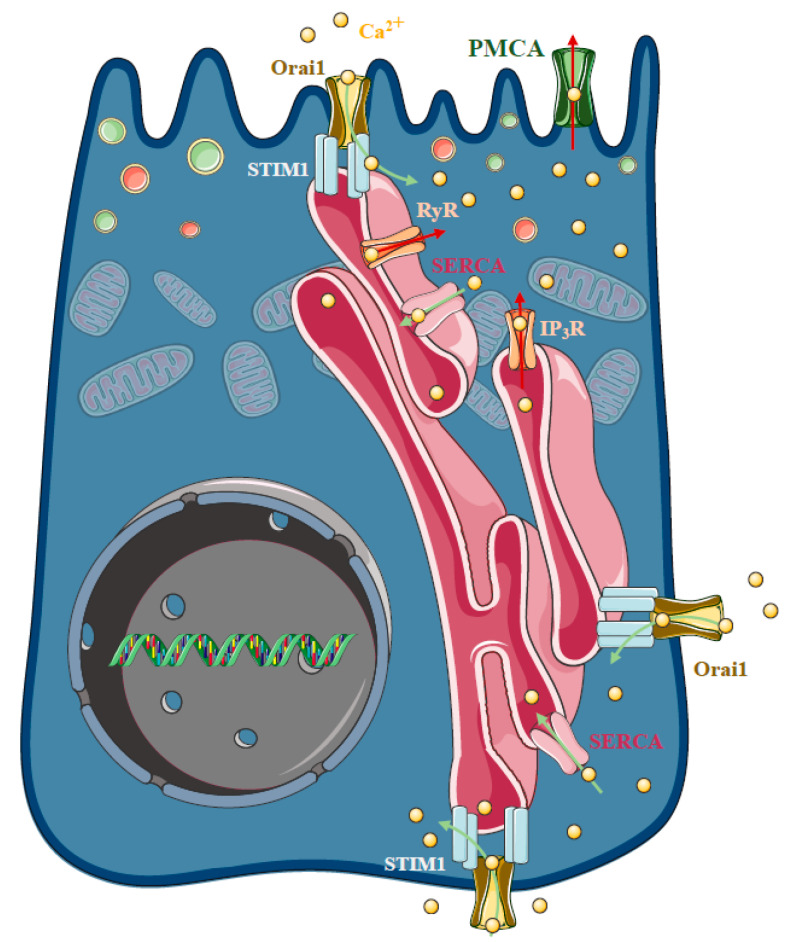
Store-operated Ca^2+^ entry and acute pancreatitis. In pancreatic acinar cells physiological agonist stimulation releases Ca^2+^ from the endoplasmic reticulum (ER) stores at the apical granular region of the cell. The spatiotemporal localisation of IP_3_-evoked apical signals is maintained by the mitochondria surrounding the apical region of the acinar cells, whereas the plasma membrane Ca^2+^ ATPase (PMCA) pumps extrude the Ca^2+^ at the apical membrane. The influx of extracellular Ca^2+^ in non-excitable cells is mediated by a process called store-operated Ca^2+^ entry (SOCE), which is determined by the ER Ca^2+^ sensor stromal interaction molecule 1 (Stim1) and the plasma membrane Ca^2+^ channel Orai1. In unstimulated cells, Stim1 is distributed in the ER membrane, whereas mobilisation of the ER Ca^2+^ stores induces a conformational change and translocation with puncta formation, of Stim1 to the ER–PM junctions, which activates Orai1. The basal Ca^2+^ uptake can refill the apical Ca^2+^ stores by a mechanism called Ca^2+^ tunnelling. Under pathological conditions, the spatiotemporal regulation of the Ca^2+^ signalling fails and extracellular Ca^2+^ entry is an important contributor to the Ca^2+^ toxicity. Recently, an Orai1 channel regulator protein called store-operated calcium entry-associated regulatory factor (Saraf) was described as a crucial component in pathological Ca^2+^ signal development. Saraf knockout mice developed more severe acute pancreatitis (AP) compared to controls accompanied by increased Ca^2+^ influx in acinar cells, whereas Saraf overexpression reduced acinar Ca^2+^ influx and decreased the severity of AP.

As highlighted above, sustained intracellular Ca^2+^ elevation plays a central role in the development of cellular injury in AP, regardless of the ethiology. In the polarised pancreatic acinar cells, the release of the ER Ca^2+^ stores in response to physiological agonist stimulation takes place at the apical granular region of the cell [63,64] (Figure 4). This apical release is achieved by the invasion of ER into the granular pole surrounding zymogen granules [65]. The spatiotemporal localisation and thus prevention of the evolution of local IP_3_-evoked signals to global intracellular Ca^2+^ elevation is prevented by the mitochondria surrounding the apical region of the acinar cells [66], whereas the plasma membrane Ca^2+^ ATPase (PMCA) pumps extrude the Ca^2+^ at the apical membrane [67]. The release of the ER Ca^2+^ stores activates the influx of extracellular Ca^2+^, which is mediated by store operated Ca^2+^ entry (SOCE), a process determined by the ER Ca^2+^ sensor Stim1 [68] and the plasma membrane Ca^2+^ channel Orai1 [69,70]. In unstimulated cells, Stim1 is distributed in the ER membrane, whereas the ER Ca^2+^ store mobilisation induces a conformational change and translocation (puncta formation) of Stim1 to the ER–PM junctions [71], leading to the activation of Orai1 and members of the TRPC channel family [72,73,74,75,76]. Mogami et al. provided direct functional evidence that Ca^2+^ entry can occur through the basal membrane of the pancreatic acinar cells [77]. In these series of elegant experiments, basal Ca^2+^ entry was controlled with a Ca^2+^-containing pipette attached to the basal membrane in extracellular Ca^2+^-free media. After recharging the intracellular Ca^2+^ stores, repeated administration of ACh could, again, trigger an increase in intracellular Ca^2+^ concentration, starting at the apical secretory pole. These results suggest that the recharging of the apical ER Ca^2+^ stores depends on Ca^2+^ influx through the basal membrane. However, the cytosolic diffusion of Ca^2+^ from the basolateral to the apical pole could have potential unwanted effects. Therefore, the concept of Ca^2+^ tunneling through the lumen of the ER has been developed, which suggests that Ca^2+^ can diffuse more easily within the ER lumen compared to the cytosol [78,79]. In isolated pancreatic acinar cells, Orai1 expression has been described independently by two different groups. Lur et al. demonstrated that stimulation-induced Stim1 translocation occurs in the lateral and basal plasma membranes of acinar cells by utilising ribosome-free terminals of the ER, which form junctions between the ER and the plasma membrane [80]. Interestingly, and somewhat contradictorily, another study by Hong et al. showed that Orai1 expression is more pronounced in the apical membrane [81]. The authors also highlighted that agonist stimulation induced polarised recruitment of Stim1 to the apical and lateral regions, showing approximately 40% colocalisation with Orai1. The authors found that both Orai1 and TRPC channels contributed to the frequency of Ca^2+^ oscillations in acinar cells.

As highlighted, the release of the ER Ca^2+^ stores activates the influx of extracellular Ca^2+^, which, under physiological conditions, helps to maintain secretory responses. However, the presence of different toxins, which damage mitochondria, lead to the impairment of ATP-dependent Ca^2+^ extrusion by PMCA as well as Ca^2+^ reuptake by SERCA [21]. As a result, the spatiotemporal localisation of the Ca^2+^ signal is lost and a global sustained Ca^2+^ elevation is developed. The pathological role of Orai1 in pancreatic acinar cells has been highlighted by Gerasimenko et al., who showed that the inhibition of SOCE via Orai1 decreases acinar cell necrosis in vitro [82]. The authors used GSK-7975A, a Ca^2+^ release-activated Ca^2+^ (CRAC) channel blocker developed by GlaxoSmithKline, which inhibits SOCE in a concentration-dependent manner and reduces the sustained Ca^2+^ elevation, trypsin activation, and acinar necrosis induced by FAEE. Moreover, the inhibition of Orai1 by selective inhibitors, GSK-7975A and CM-128 by CalciMedica, markedly impaired the extracellular Ca^2+^ influx and sustained Ca^2+^ overload in pancreatic acinar cells upon bile acid stimulation, significantly reducing pancreatic edema, inflammation and necrosis in experimental models of AP [83]. Using CM4620, another selective Orai1 channel blocker by CalciMedica, Waldron et al. demonstrated that the inhibition of SOCE prevents trypsinogen activation, acinar cell death, NF-κB and nuclear factor of activated T-cells (NFAT) activation, inflammatory responses in in vitro models, and decreases the severity of experimental AP in mice [84]. In addition, they also showed that CM4620 abolished myeloperoxidase activity and inflammatory cytokine expression in pancreas and lung tissues and prevented the oxidative burst in neutrophils. In cerulein-treated mice, cerulein activates SOCE through a promotion of the interaction between Stim1 and Orai1. Intracellular Ca^2+^ elevation, induced by SOCE-activated NFAT and transcription factor EB, led to the calcineurin-promoting transcription of chemokine genes and autophagy-associated genes [85]. Orai1 was also identified as a central molecule in regulation of the gut microbiome and host immune system [86]. Orai1 pancreatic acinar conditional knockout mice display intestinal bacterial outgrowth and dysbiosis, leading to systemic translocation, inflammation and remarkably reduced survival, all of which were due to the decreased pancreatic levels of cathelicidin-related antimicrobial peptide. This was markedly improved by treatment with a liquid diet and broad-spectrum antibiotics, which rescued weight gain and survival.

On the other hand, genetic deletion of TRPC3 was found to markedly reduce receptor-stimulated SOCE by about 50% and prevent sustained Ca^2+^ elevation triggered by the bile acids and ethanol metabolites. TRPC3 deletion also prevented intracellular trypsin activation and inhibition of digestive enzyme secretion. These beneficial effects led to reduced severity of cerulein-induced AP in vivo [87]. The same protection was achieved by Pyr3, a TRPC3-specific inhibitor [88].

Although Orai1 inhibition seems to be beneficial in AP, this channel is also necessary for T cell activation [89]. Therefore, the identification of other potential target proteins is desirable. Recently, an Orai1 channel regulator protein, called store-operated calcium entry-associated regulatory factor (Saraf) [90], was described as a crucial component in pathological Ca^2+^ signal development [91]. In contrast to Stim1 and Orai1 expression, which were not changed during AP, Saraf expression was decreased during AP in mice and humans. In addition, Saraf knockout mice developed more severe AP compared to controls, which was accompanied by increased Ca^2+^ influx in acinar cells. Saraf overexpression reduced acinar Ca^2+^ influx and decreased the severity of AP. These results highlight the crucial role of Saraf in AP pathogenesis and identify this regulatory protein as potential target for therapy.

## 4. Ca^2+^ Signalling in Pressure-Related Acute Pancreatitis

**Figure 5:**
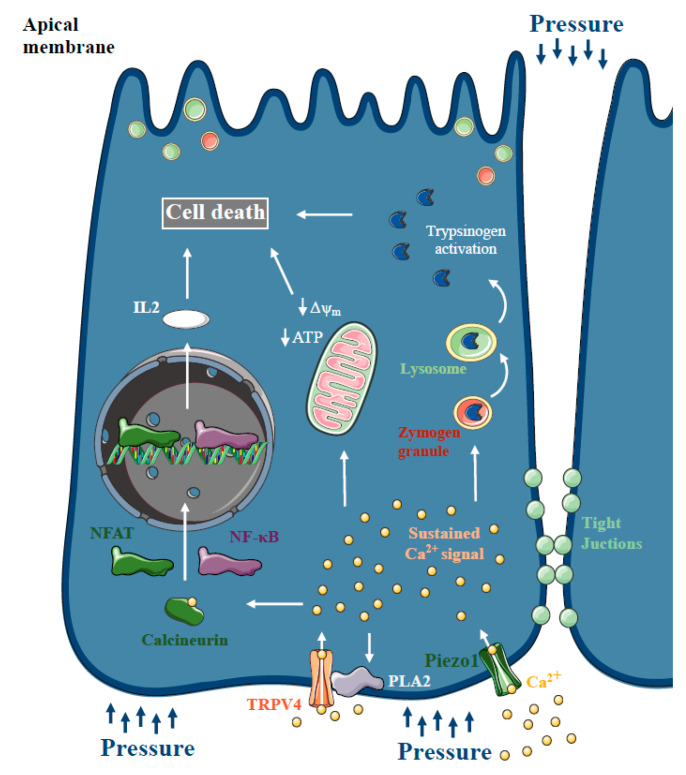
Ca^2+^ signalling in pressure-related acute pancreatitis. Increased intrapancreatic pressure during endoscopic retrograde cholangiopancreatography (ERCP) can damage the pancreas and cause post-ERCP acute pancreatitis (AP). Similar to other forms of AP, pancreatic acinar cells represented aberrant intracellular Ca^2+^ signalling and impaired mitochondrial function and calcineurin downstream activation. Recently, Piezo1 expression, a mechanoreceptor directly gated by mechanical forces, was described in pancreatic acinar cells. Piezo1 activation triggered intracellular Ca^2+^ elevation and acinar cell injury. Genetic deletion of Piezo1 remarkably decreased the severity of pressure-induced AP, and it’s activation by Yoda1 triggered AP without the application of pressure. In addition, Piezo1 activation induces phospholipase A2 activation, which activates TRPV4, which is necessary for the development of sustained toxic Ca^2+^ signals in acinar cells upon pressure.

For several decades, there has been a theory that increased intrapancreatic pressure can lead to pancreatic damage. Post-ERCP pancreatitis is one of the most common forms of iatrogenic AP, with a 10% overall incidence and a 0.7% mortality rate [92]. Post-ERCP AP is thought to be provoked by increased intrapancreatic pressure and intraductal hypertension, in combination with the acidic radiocontrast solution. Using American opossum as an experimental model of pressure-induced pancreatitis, Lerch et al. provided evidence that pancreatic outflow obstruction itself, without the presence of bile acids, is sufficient to trigger necrotising AP [93,94]. Within 6 h of pancreatic and bile duct ligation, the animals developed transient pancreatic edema and progressive hyperamylasaemia, followed by acinar cell necrosis, haemorrhage, fat necrosis and inflammatory cell infiltration, within 12 h of duct obstruction. Notably, in other species, duct ligation alone induces only mild pancreatitis, rather than severe necrotising AP, except in the American opossum [95]. Studies including human patients have also confirmed that transient obstruction of the pancreatic duct can lead to AP [96,97]. These observations, and the fact that secretory pressure in the pancreatic duct is generally much higher than in the bile duct [98,99], also lead to the criticism of the common channel formation hypothesis and bile acid reflux during biliary pancreatitis [100]. More recently, Jin et al. demonstrated that intracellular Ca^2+^ signalling plays a central role in the pathogenesis of pressure-related pancreatic injury. They showed that incubation of mouse and human acinar cells with radiocontrast solutions triggered sustained intracellular Ca^2+^ elevation and activated the transcription factors NF-κB and NFAT via calcineurin. Genetic or pharmacologic inhibition of calcineurin prevented radiocontrast-induced pancreatic inflammation in mice. In addition, Wen et al. demonstrated that artificially increased intraductal pressures (100–150 mmHg for 10 min) are sufficient to trigger AP accompanied by increased serum amylase, cytokine release and tight junction integrity loss [101]. Similar to the previous observations, pancreatic acinar cells exhibited aberrant intracellular Ca^2+^ signalling, impaired mitochondrial function and calcineurin downstream activation (Figure 5). More mechanistic insight was provided into the development of pressure-induced pancreatic damage by Romac et al. [102]. The authors demonstrated that Piezo1, a mechanoreceptor directly gated by mechanical forces [103], is expressed in pancreatic acinar cells; application of pressure within the gland leads to AP through Piezo1 activation. Yoda1 administration, a Piezo1 agonist, triggered intracellular Ca^2+^ elevation and acinar cell injury, including mitochondrial depolarisation, trypsinogen activation and acinar cell necrosis, which was abolished by specific inhibition or genetic dysruption of Piezo1. In addition, genetic deletion of Piezo1 remarkably decreased the severity of pressure-induced AP, whereas Piezo1 activation by Yoda1 triggered AP without the application of pressure. In another study by the same group, they showed that mechanical pushing and fluid shear stress increase the intracellular Ca^2+^ levels in pancreatic acini [104]. They also provided evidence that Yoda1 administration induces phospholipase A2 activation, which activates TRPV4. They concluded that TRPV4 activation is necessary for the development of sustained toxic Ca^2+^ signals in acinar cells upon pressure.

Although intrapancreatic pressure elevation also affects pancreatic ductal function, none of these studies assessed ductal secretion [105]. Alkaline secretion of the ductal cells washes out digestive proenzymes into the duodenum and the alkaline fluid neutralises the protons co-released during digestive enzyme secretion by pancreatic acinar cells [106]. Increased intraluminal pressure in the pancreatic ducts was shown to inhibit pancreatic secretion in vitro and in vivo via a serotonin-mediated pathway, which involves the activation of 5-HT_3_ receptors in the duct cells [107]. In addition, the pancreatic fluid and HCO_3_^-^ secretion is significantly elevated in the absence of peripheral serotonin [108]. The alkaline intraductal pH also prevents premature trypsinogen activation, as the autoactivation of trypsinogen is a pH-dependent process, which is accelerated at acidic pH ranges. Impaired ductal secretion in CFTR knockout mice alters the membrane dynamics and endocytosis at the apical plasma membrane of pancreatic acinar cells [109]. Decreased ductal secretion was described in Na^+^/H^+^ exchanger regulatory factor-1 knockout mice due to the loss of CFTR, leading to more severe experimental AP [110]. Importantly, pharmacological restoration of CFTR function in pancreatic ducts corrected acinar cell function and decreased pancreatic inflammation [111]. Therefore, maintaining physiological ductal function and the alkaline luminal pH undoubtedly protects the pancreas from autodigestion [112]. Based on these findings, we hypothesise that the ductal cells play a significant part in the development of pressure-related pancreatic injuries, which will need to be further examined in future studies.

## 5. Translational Perspectives

Inflammatory disorders of the pancreas, such as acute and chronic pancreatitis, pose a significant clinical challenge, as there are currently no specific pharmaceutical treatments available. As discussed in detail above, basic science studies can identify pathogenic disease mechanisms and novel drug targets, which can support drug discovery and therapy development in pancreatic diseases. Disturbed intracellular Ca^2+^ signalling is a well-established hallmark of the disease, which induces increased ROS production, mitochondrial damage, intra-acinar digestive enzyme activation, and cell death. Due to this mechanism of action, preventing toxic cellular Ca^2+^ overload is currently one of the most promising therapeutic targets [113]. SOCE inhibition via Orai1 channels improved several key markers of AP in vitro and in vivo, as discussed above. Therefore, small-molecule inhibitors of Orai1 channel seem to be one of the most promising drug candidates to treat AP and its complications. CalciMedica has successfully completed a Phase I clinical trial enrolling seven patients with AP to assess the pharmacokinetic and pharmacodynamics profiles of the CM4620 Orai1 channel inhibitor [114]. As a next step, the company is currently running a Phase II trial (an open-label, dose-response study) to evaluate the safety and efficacy of CM4620-IE in patients with AP and accompanying systemic inflammatory response syndrome (SIRS) [115].

Our recent study identified TRPM2 as an important contributor to oxidative-stress-induced cellular Ca^2+^ overload in biliary AP. TRPM2 activation contributed to bile-acid-induced extracellular Ca^2+^ influx in acinar cells, which promoted necrosis in vitro and in vivo. In an experimental model of biliary AP, genetic knockout of TRPM2 significantly decreased the disease severity and protected acinar cells. Based on these results, TRPM2 inhibition may be a potential treatment option for biliary pancreatitis and development of novel TRPM2 inhibitors can be translated to patients. In a recent study, Fourgeaud et al. identified JNJ-28583113 as a potent selective inhibitor of TRPM2 [116]. They demonstrated that JNJ-28583113 caused phosphorylation of GSK3α and β subunits of TRPM2 in nM concentrations, which protected cells from oxidative stress-induced cell injury and impaired cytokine release in response to pro-inflammatory stimuli in microglia. Although JNJ-28583113 was promising in in vitro experiments, in vivo administration is currently limited by the rapid metabolisation of the drug. TRPM2 has been identified as a promising drug target in diseases of the central nervous system, such as neuropathic pain, bipolar disorder and Alzheimer’s disease, and more potential inhibitors are expected to be identified.

Toxin-induced mitochondrial injury is another key pathogenic step of AP development; therefore, protection of mitochondrial ATP production is another potential target [117]. Maintenance of ATP production could provide the necessary ATP to fuel ATP-dependent Ca^2+^ extrusion mechanisms, such as SERCA and PMCA pumps, which ensures the spatiotemporal regulation of Ca^2+^ signals. Opening of the MPTP, which is a non-specific channel that forms in the inner mitochondrial membrane allowing passage of particles under 1500 Da, causes loss of the ΔΨ_m_ that is essential to ATP production [118]. MPTP inhibition in pancreatic acinar cells successfully improved the outcome of AP in mice models [16]. Our group also demonstrated that a novel mitochondrial transition pore inhibitor, *N*-methyl-4-isoleucine cyclosporin (NIM811), decreases the severity of AP [17]. We demonstrated that NIM811 administration, an MPTP blocker that acts on cyclophilin D, protects acinar and ductal cells from bile acid and fatty acid exposure in vitro. NIM811 decreases the severity of AP in vivo in cerulein, bile acid and fatty acid-induced AP models, making NIM811 a promising candidate for further development.
